# Genetic variants in autophagy-related gene *ATG2B* predict the prognosis of colorectal cancer patients receiving chemotherapy

**DOI:** 10.3389/fonc.2022.876424

**Published:** 2022-08-05

**Authors:** Ting Yu, Shuai Ben, Ling Ma, Lu Jiang, Silu Chen, Yu Lin, Tao Chen, Shuwei Li, Lingjun Zhu

**Affiliations:** ^1^Department of Oncology, The First Affiliated Hospital of Nanjing Medical University, Nanjing, China; ^2^Department of Genetic Toxicology, The Key Laboratory of Modern Toxicology of Ministry of Education, Center for Global Health, School of Public Health, Nanjing Medical University, Nanjing, China; ^3^Department of Environmental Genomics, Jiangsu Key Laboratory of Cancer Biomarkers, Prevention and Treatment, Collaborative Innovation Center for Cancer Personalized Medicine, Nanjing Medical University, Nanjing, China; ^4^Department of Gastrointestinal Surgery, The First Affiliated Hospital of Nanjing Medical University, Nanjing, China; ^5^Department of Oncology, Sir Run Run Hospital, Nanjing Medical University, Nanjing, China

**Keywords:** colorectal cancer, ATG2B, genetic variants, chemotherapy, prognosis

## Abstract

Autophagy-related genes have a vital effect on colorectal cancer (CRC) by affecting genomic stability and regulating immune responses. However, the associations between genetic variants in autophagy-related genes and CRC outcomes for chemotherapy therapy remain unclear. The Cox regression model was used to evaluate the associations between single-nucleotide polymorphisms (SNPs) in autophagy-related genes and overall survival (OS) and progression-free survival (PFS) of CRC patients. The results were corrected by the false discovery rate (FDR) correction. We used the logistic regression model to investigate the associations of SNPs with the disease control rate (DCR) of patients. Gene expression analysis was explored based on an in-house dataset and other databases. The associations between gene expression and infiltrating immune cells were evaluated using the Tumor Immune Estimation Resource (TIMER) database. We observed that *ATG2B* rs17094017 A > T was significantly associated with increased OS (HR = 0.65, 95% CI = 0.50-0.86, *P* = 2.54×10^-3^), PFS (HR = 0.76, 95% CI = 0.62-0.93, *P* = 7.34×10^-3^), and DCR (OR = 0.60, 95% CI = 0.37-0.96, *P* = 3.31×10^-2^) of CRC patients after chemotherapy. The expression of *ATG2B* was down-expressed in CRC tissues than in adjacent normal tissues. Moreover, *ATG2B* expression influenced the infiltration of CD8+ T cells, CD4+ T cells, B cells, and T cell receptor signaling pathways, which may inhibit the occurrence of CRC by affecting the immune system. This study suggests that genetic variants in the autophagy-related gene *ATG2B* play a critical role in predicting the prognosis of CRC prognosis undergoing chemotherapy.

## Introduction

Colorectal cancer (CRC) is regarded as the third-leading cause of morbidity, as well as the third-leading cause of fatality rates in the United States (1). By 2030, the mortality of individuals with CRC will increase by more than 1.1 million worldwide, while the number of CRC cases will exceed 2.2 million (2). In China, CRC ranks the fifth in cancer‐related mortality and the second in cancer incidence (3). Several risk factors for CRC were identified by epidemiology, such as sex, age, smoking, BMI, heavy drinking, and red and processed meat (4–6). Additionally, the relationships between genetic factors and CRC risk were also verified (7). Chemotherapy, as an effective therapy for CRC, is typically used for treatment after tumor resection. Based on previous studies, a combination of leucovorin and 5-fluorouracil with either irinotecan or oxaliplatin, the first-line treatment regimen, is widely accepted therapy to treat metastatic colorectal cancer (mCRC) (8).

Autophagy is a cellular process in which macromolecules, metabolites, and damaged organelles are transported into lysosomes, where they are degraded and produced as energy materials for cell reuse. Autophagy is commonly subdivided into three types based on its mechanism: macroautophagy, microautophagy, and chaperone-mediated autophagy (9). Autophagy is of great importance in regulating cancer cell metabolism, proliferation, and survival (10, 11). Numerous studies have suggested that autophagy-related genetic variants could accelerate cancer progression (12). The effects of single nucleotide polymorphisms (SNPs) in autophagy-related genes and the outcomes and risk of breast cancer, bladder cancer, non-small cell lung cancer, CRC, and esophageal squamous cell carcinoma were reported (13–18). Recently, autophagy-related genes influence the immune response and the efficacy of immunotherapy by modulating immune system components (19). Autophagy-related genes also have an effect on radiotherapy in CRC cells (20). Furthermore, a survey conducted by Berger et al. (21) demonstrated that SNPs in autophagy-related genes contributed to the occurrence of adverse effects on chemotherapy drugs for CRC.

However, there are no related studies regarding the relationships between genetic variants in autophagy-related genes and the response to CRC chemotherapy in the Chinese population. In view of the evidence that autophagy-related genes can affect cancer prognosis, we assumed that autophagy-related gene variants were related to CRC survival after chemotherapy.

## Materials and methods

### Study populations

A cohort of 344 CRC patients who underwent histopathological examination was included in the Affiliated Nanjing First Hospital and the First Affiliated Hospital of Nanjing Medical University in September 2010. According to our follow-up data, 19 patients who did not receive oxaliplatin or irinotecan-based therapy were excluded, and 325 patients were retained. Briefly, 188 CRC patients were treated with oxaliplatin-based regimens, and 137 patients underwent irinotecan-based regimens. A total of 325 CRC patients were followed up using telephone interviewing methods. The deadline for follow-up was April 2, 2016. Details of the study population have been demonstrated (22). Overall survival (OS) after chemotherapy as the primary endpoint was calculated from the time of the first chemotherapy until death or last follow-up for living patients. Additionally, progression-free survival (PFS) refers to the day elapsed from the day of chemotherapy initiation to the day of objective disease progression, death, or last follow-up. OS, PFS, and responses to chemotherapy were considered as outcomes. Peripheral venous blood was collected with written consent for each sample (5 mL). The Institutional Review Board of Nanjing Medical University authorized our research.

### Clinical assessment of CRC patients

To assess CRC prognosis before therapy and after a minimum of two cycles of treatment, we used computed tomography as the detection method. Tumor responses to chemotherapy were regarded as the primary endpoint using Response Evaluation Criteria in Solid Tumors (RECIST 1.1). The prognosis and responses to chemotherapy were assessed by the complete response (CR), partial response (PR), progressive disease (PD), and stable disease (SD). The disease control rate (DCR) consisted of CR, PR, and SD.

### Autophagy-related genes and SNP selection

**Figure 1:**
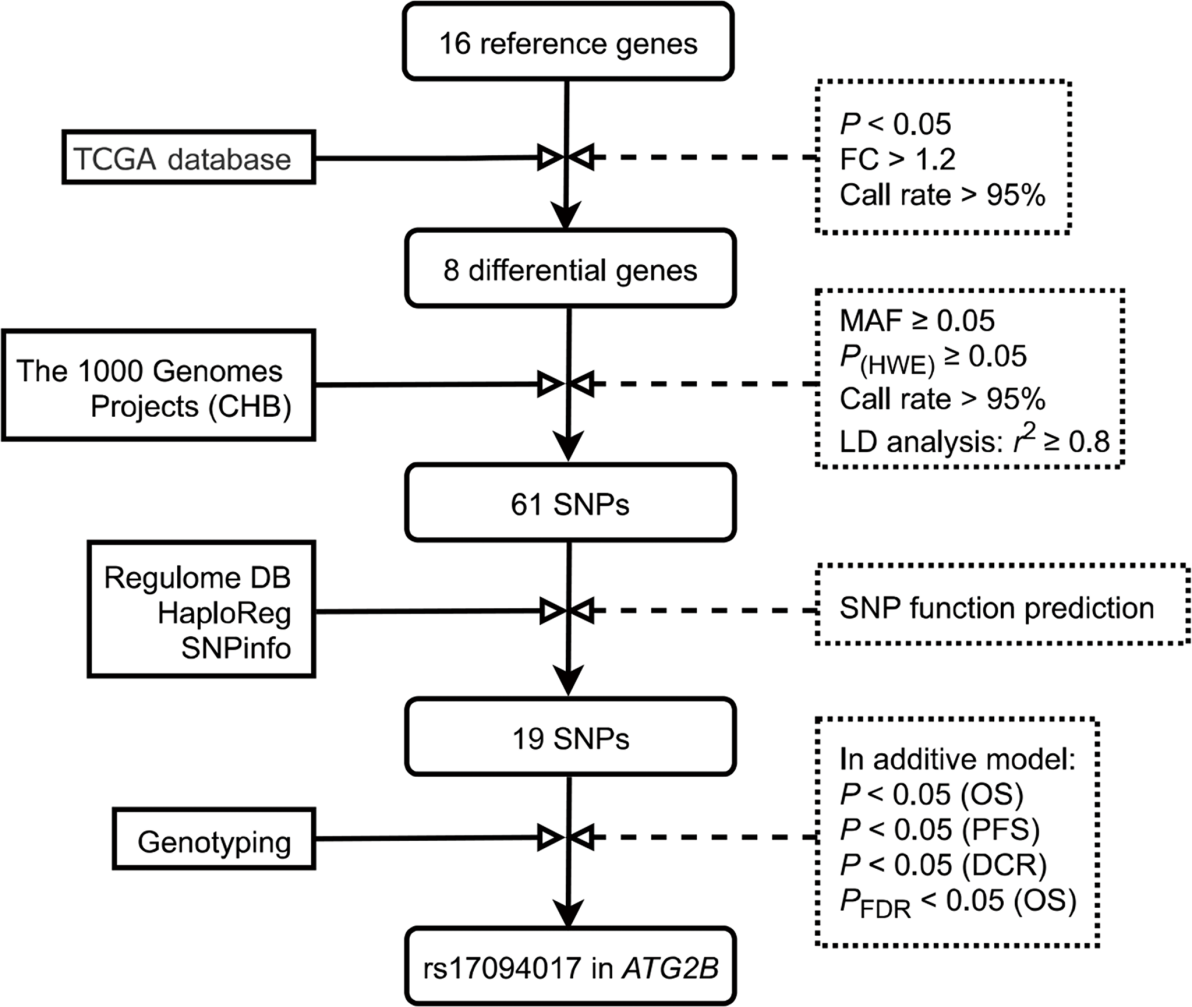
Flow diagram of SNP selection in the autophagy-related genes. First, 16 candidate genes were selected in this study. Then, 8 differentially expressed autophagy-related genes were selected by these conditions: **(A)** fold change > 1.2, **(B)** *P* < 0.05 and **(C)** call rate > 95%. SNPs within 2 kb up- and down-stream regions of 8 candidate genes were extracted using the Han Chinese in Beijing (CHB) data from the 1000 Genomes Project (March 2012) based on these selection conditions: (a) minor allele frequency (MAF) in population ≥ 0.05, (b) Hardy-Weinberg equilibrium (HWE) ≥ 0.05, (c) call rate > 95%. Tagging SNPs were recognized after linkage disequilibrium (LD) (*r*^2^ ≥ 0.8). Next, using SNPinfo Web Server, HaploReg, and RegulomeDB to predict functional SNPs. The associations between 19 SNPs and OS of CRC were assessed in the additive model, and SNPs of which adjusted *P*_FDR_ (OS) value was < 0.05 were selected. Finally, the effects of the remaining SNPs on PFS and DCR were analyzed in the additive model, and SNPs that were statistically related to PFS and DCR were selected. TCGA, The Cancer Genome Atlas; MAF, minor allele frequency; HWE, Hardy‐Weinberg equilibrium; LD, linkage disequilibrium; CHB, the Han Chinese in Beijing; SNP, single nucleotide polymorphism; PFS, progression-free survival; DCR, disease control rate; OS, Overall survival; FDR, false discovery rate.

Reactome, and Kyoto Encyclopedia of Genes and Genomes were applied to select autophagy-related genes. To find autophagy-related genes more comprehensively, we systematically searched the keywords ‘cancer’, ‘carcinoma’, ‘tumor’, ‘autophagy’, and ‘autophagy-related genes’ in PubMed. In our study, to exclude the effect of gender, we eliminated the genes located on the X chromosome. Briefly, we selected 16 candidate genes for further analysis. We compared the gene expressions between CRC tissues and normal tissues, and genes were selected by these gene screening conditions: (a) fold change > 1.2, (b) *P* < 0.05 and (c) call rate > 95%. Finally, 8 differentially expressed autophagy-related genes were found in CRC tissues and normal tissues for further study. The schematic diagram of SNP selection was presented in **Figure 1**. The flow diagram of SNP selection was similar to a previous study (23). Firstly, we extracted SNPs within 2 kb up- and down-stream regions of 8 differentially expressed autophagy-related genes using the Han Chinese in Beijing (CHB) data from the 1000 Genomes Project (March 2012) based on these selection conditions: (a) minor allele frequency (MAF) in population ≥ 0.05, (b) Hardy-Weinberg equilibrium (HWE) ≥ 0.05, (c) call rate > 95%. Secondly, SNPinfo Web Server (24), HaploReg (25), and RegulomeDB (26) were used to predict functional SNPs. SNPs were not included when the RegulomeDB score > 6. Thirdly, we selected tagged SNPs after linkage disequilibrium (LD) (*r*^2^ ≥ 0.8) by PLINK 1.09 (27). A total of 19 SNPs were retained in our study. Fourthly, the associations between 19 SNPs and the OS of CRC were assessed in the additive model. The results were corrected by the false discovery rate (FDR), and we selected SNPs of which adjusted *P*_FDR_ (OS) value was < 0.05. Finally, the effects of the remaining SNPs on PFS and DCR were analyzed in the additive model, and SNPs that were statistically related to PFS and DCR were selected.

### SNP genotyping

To extract genomic DNA from blood samples of CRC patients, we used the Qiagen Blood Kit (Qiagen). The method of extracting genomic DNA was the same as in a previous study (23). For genotyping in this study, Illumina Human Omni Zhonghua Bead Chips were employed. The microarray is a genome-wide SNP genotyping microarray designed specifically for Chinese people. The microarray covers optimized 900,000 labeled common and rare variants found in the Chinese population, which are strategically selected to explore novel disease and trait associations in the Chinese population. A uniform quality control protocol was used to filter samples and SNPs.

### Expression analyses

We compared the expression of candidate genes in CRC tissues and noncancerous tissues using the mRNA expression data from the Gene Expression Omnibus (GEO) dataset (GSE87211), the Cancer Genome Atlas (TCGA) database and in-house RNA-Seq data. To assess targeted gene expression among various subtypes of CRC, we applied the Tumor and Immune System Interaction Database (TISIDB) (http://cis.hku.hk/TISIDB/index.php). We evaluated candidate gene expression in pan‐cancer with the Gene Expression Profiling Interactive Analysis (GEPIA) (http://gepia.cancer-pku.cn/). To confirm the protein level of ATG2B, we used the Human Protein Atlas (HPA) database (https://www.proteinatlas.org/) and the Clinical proteomic tumor analysis consortium (CPTAC) dataset.

### Functional analyses

The protein-protein interaction network with autophagy-related genes was explored using the STRING database (https://string-db.org/). The volcano plot was performed to demonstrate differentially expressed genes (DEGs) in high- and low-expressed *ATG2B* groups. Biological pathways and functions within the subgroups of down-regulated and up-regulated *ATG2B* expression were assessed using Gene Set Enrichment Analysis (GSEA). Using the Tumor Immune Estimation Resource (TIMER) database (https://cistrome.shinyapps.io/timer/) to investigate the roles of *ATG2B* expression in immune infiltration.

### Statistical analyses

Statistical methods with univariate Cox regression analysis were utilized to assess associations between the clinical characteristics and OS of CRC patients (24). The results were corrected by the FDR, which was employed to mitigate against false-positive results. The associations between targeted SNPs and CRC prognosis were assessed depending on multivariate and univariate COX regression models (24). Logistic regression models were employed to identify the effects of SNPs on DCR. The odds ratios (ORs), hazard ratios (HRs), and 95% confidence intervals (CIs) were calculated for the genetic variants. A two-sided Student’s *t* test was utilized to analyze the differential expression of genes. A Kaplan-Meier analysis was applied to estimate the survival probability. *P* values below 0.05 were considered significant. All statistical computations were achieved by R 3.2.3 and PLINK 1.09.

## Results

### Subject characteristics

Clinical characteristics of 325 CRC patients and their associations with OS were presented in **Supplementary Table 1**. However, no significant difference was discovered between these characteristics and OS (*P* > 0.05). In our study, 205 (63.08%) CRC patients were males, and 120 (36.92%) were females; 59.69% of patients were diagnosed with colon cancer. Patients with poorly differentiated tumors accounted for 21.54% of the total, and 78.46% of CRC patients were in intermediate or advanced tumor stages. Moreover, 7.08% of patients had Dukes stage C disease, and 92.92% were in Dukes stage D.

### SNP selection and the genetic effect of SNPs in *ATG2B* on CRC

A total of 16 genes were ultimately selected in our research (**Supplementary Table 2**). The interactions among these proteins were presented in **Supplementary Figure 1**. Based on the TCGA database, we selected 8 differentially expressed autophagy-related genes in CRC tissues and normal tissues for further study (**Supplementary Table 3**). After quality control and LD analysis, a total of 61 SNPs remained. Then, using RegulomeDB, HaploReg, and SNPinfo Web Server to annotate the potential functions of SNPs, we selected 19 SNPs for further analysis (**Supplementary Table 4**).

### The association between rs17094017 in *ATG2B* and CRC prognosis

A total of 19 candidate SNPs were evaluated for associations with the OS of CRC in the additive model. The results illustrated significant associations between rs17094017 and rs11658979 and the OS of CRC (*P* < 0.05) ( **Table 1**). After FDR correction, only *ATG2B* rs17094017 was significantly associated with a favorable OS (*P*_FDR_ = 4.83×10^-2^) of CRC. Then, we analyzed whether candidate SNPs were relevant to PFS and DCR in CRC patients. In agreement with previous results, rs17094017 prolonged the PFS (HR = 0.76, 95% CI = 0.62-0.93, *P* = 7.34×10^-3^) and increased the DCR (OR = 0.60, 95% CI = 0.37-0.96, *P* = 3.31×10^-2^) of CRC (**Supplementary Table 5**).

**Table 1:** Association between selected 19 SNPs and overall survival of clinical patients with CRC.

Chr	SNP	Gene	Position^a^	MAF^b^	Allele^c^	OS (Overall survival)				
						HR (95% CI)	*P*	HR (95% CI)^d^	*P* ^d^	*P* ^e^
2	rs35271226	*ATG4B* intron	242580470	0.45	A/G	1.18 (0.94-1.49)	1.52×10^-1^	1.22 (0.96-1.53)	1.01×10^-1^	4.10×10^-1^
2	rs1130910	*ATG4B* intron	242611050	0.16	G/C	1.16 (0.88-1.53)	3.00×10^-1^	1.23 (0.93-1.64)	1.49×10^-1^	4.71×10^-1^
2	rs7421	*ATG4B* 3’-UTR	242611934	0.45	C/T	1.15 (0.90-1.47)	2.60×10^-1^	1.12 (0.88-1.43)	3.57×10^-1^	5.39×10^-1^
2	rs6758317	*ATG16L1* intron	234168951	0.11	T/C	0.82 (0.54-1.25)	3.58×10^-1^	0.83 (0.54-1.25)	3.69×10^-1^	5.39×10^-1^
2	rs34691302	*ATG4B* intron	242577665	0.09	T/C	1.00 (0.64-1.56)	9.93×10^-1^	1.07 (0.68-1.69)	7.56×10^-1^	9.06×10^-1^
2	rs2241878	*ATG16L1* intron	234183718	0.37	C/T	1.01 (0.80-1.28)	9.10×10^-1^	1.04 (0.82-1.30)	7.63×10^-1^	9.06×10^-1^
2	rs7595748	*ATG16L1* intron	234193186	0.46	A/G	1.01 (0.80-1.27)	9.44×10^-1^	0.98 (0.78-1.23)	8.66×10^-1^	9.15×10^-1^
14	rs17094017	*ATG2B* intron	96783727	0.23	T/A	0.65 (0.50-0.85)	1.82×10^-3^	0.65 (0.50-0.86)	2.54×10^-3^	4.83×10^-2^
14	rs8019013	*ATG2B* 3’-UTR	96751010	0.49	T/C	1.22 (0.96-1.56)	1.06×10^-1^	1.22 (0.96-1.56)	1.08×10^-1^	4.10×10^-1^
14	rs12432561	*ATG2B* intron	96762271	0.24	A/G	1.08 (0.83-1.40)	5.66×10^-1^	1.06 (0.82-1.38)	6.43×10^-1^	8.73×10^-1^
14	rs10134160	*ATG2B* 3’-UTR	96747986	0.17	T/C	0.97 (0.70-1.33)	8.29×10^-1^	0.97 (0.70-1.33)	8.43×10^-1^	9.15×10^-1^
16	rs11149841	*GABARAPL2* intron	75602797	0.08	T/G	0.79 (0.48-1.28)	3.30×10^-1^	0.79 (0.48-1.28)	3.32×10^-1^	5.39×10^-1^
16	rs6564267	*GABARAPL2* intron	75603925	0.09	T/G	0.98 (0.64-1.49)	9.11×10^-1^	0.99 (0.64-1.51)	9.53×10^-1^	9.53×10^-1^
17	rs11658979	*WIPI1* intron	66419229	0.14	G/A	1.47 (1.04-2.09)	3.01×10^-2^	1.47 (1.03-2.09)	3.30×10^-2^	3.14×10^-1^
17	rs11077558	*WIPI1* intron	66428002	0.35	C/G	1.16 (0.92-1.46)	2.00×10^-1^	1.22 (0.97-1.54)	9.33×10^-2^	4.10×10^-1^
17	rs2011143	*WIPI1* intron	66422955	0.31	T/C	1.12 (0.88-1.41)	3.62×10^-1^	1.16 (0.91-1.47)	2.31×10^-1^	5.39×10^-1^
17	rs2909207	*WIPI1* intron	66439605	0.41	T/C	1.11 (0.89-1.38)	3.65×10^-1^	1.14 (0.91-1.43)	2.48×10^-1^	5.39×10^-1^
17	rs883622	*WIPI1* intron	66442603	0.28	G/A	0.90 (0.70-1.15)	3.97×10^-1^	0.88 (0.69-1.13)	3.26×10^-1^	5.39×10^-1^
17	rs883620	*WIPI1* intron	66442130	0.05	C/G	1.21 (0.79-1.83)	3.79×10^-1^	1.22 (0.80-1.86)	3.54×10^-1^	5.39×10^-1^

Chr, chromosome; SNP, single nucleotide polymorphism; HR, hazard ratio; CI, confidence interval.

a Based on NCBI build 37 of the human genome.

b Minor allele frequencies were calculated by the in-house data.

c Effect allele/reference allele.

d *P* value for additive model adjusted for sex, age, smoking status and drinking status in Cox regression model.

e The false discovery rate correction of *P* value.

To evaluate the effects of rs17094017 on *ATG2B* and CRC survival, we performed Cox regression analyses and logistic regression analyses of four models (dominant model, additive model, codominant and recessive model). Patients with the T allele had an improved prognosis (OS: HR = 0.57, 95% CI = 0.41-0.80, *P* = 1.03×10^-3^; PFS: HR = 0.74, 95% CI = 0.56-0.96, *P* = 2.40×10^-2^) compared with those harboring the A allele in the dominant model (**Table 2**). Moreover, a similar result was found in DCR (OR = 0.55, 95% CI = 0.31-0.99, *P* = 4.43×10^-2^) (**Table 3**). In addition, TT genotype carriers had a longer PFS of CRC than AA genotype carriers in the codominant model (HR = 0.53, 95% CI = 0.33-0.87, *P* = 1.27×10^-2^) (**Table 2**). Under the recessive model, we also found that rs17094017 with improvement of PFS was significant (HR = 0.60, 95% CI = 0.37-0.96, *P* = 3.34×10^-2^) (**Table 2**). However, in the recessive model, there were no differences between rs17094017 and OS or DCR (*P* > 0.05).

**Table 2:** Association between *ATG2B* rs17094017 and the survival of clinical patients with CRC.

Genotypes	Deaths (%)	OS (overall survival)				Progress (%)	PFS (progression-free survival)			
		HR (95% CI)	*P*	HR (95% CI)^a^	*P*^a^		HR (95% CI)	*P*	HR (95% CI)^a^	*P*^a^
AA	85 (57.43)	1.00		1.00		116 (51.10)	1.00		1.00	
AT	52 (35.14)	0.58 (0.41-0.82)	1.85×10^-3^	0.58 (0.40-0.82)	2.26×10^-3^	92 (40.53)	0.77 (0.59-1.02)	6.77×10^-2^	0.80 (0.60-1.05)	1.10×10^-1^
TT	11 (7.43)	0.54 (0.28-1.01)	5.27×10^-2^	0.54 (0.29-1.03)	6.08×10^-2^	19 (8.37)	0.53 (0.32-0.86)	1.07×10^-2^	0.53 (0.33-0.87)	1.27×10^-2^
Additive model		0.65 (0.50-0.85)	1.82×10^-3^	0.65 (0.50-0.86)	2.54×10^-3^		0.75 (0.61-0.91)	4.53×10^-3^	0.76 (0.62-0.93)	7.34×10^-3^
Dominant model		0.57 (0.41-0.79)	7.57×10^-4^	0.57 (0.41-0.80)	1.03×10^-3^		0.72 (0.55-0.93)	1.36×10^-2^	0.74 (0.56-0.96)	2.40×10^-2^
Recessive model		0.69 (0.37-1.28)	2.37×10^-1^	0.70 (0.38-1.31)	2.70×10^-1^		0.60 (0.37-0.96)	3.36×10^-2^	0.60 (0.37-0.96)	3.34×10^-2^

HR, hazard ratio; CI, confidence interval. ^a^Adjusted for sex, age, smoking and drinking status in Cox regression model.

**Table 3:** Association between *ATG2B* rs17094017 and responses to chemotherapy of clinical patients with CRC.

Genotypes	PD (progress disease) (%)	DCR (disease control rate)			
		OR (95% CI)	*P*	OR (95% CI)^a^	*P* ^a^
AA	38 (59.37)	1.00		1.00	
AT	23 (35.94)	0.59 (0.33-1.06)	8.00×10^-2^	0.60 (0.33-1.09)	9.19×10^-2^
TT	3 (4.69)	0.36 (0.10-1.28)	1.15×10^-1^	0.35 (0.10-1.25)	1.07×10^-1^
Additive model		0.60 (0.38-0.95)	3.10×10^-2^	0.60 (0.37-0.96)	3.31×10^-2^
Dominant model		0.55 (0.32-0.97)	3.88×10^-2^	0.55 (0.31-0.99)	4.43×10^-2^
Recessive model		0.46 (0.13-1.58)	2.15×10^-1^	0.45 (0.13-1.55)	2.03×10^-1^

OR, odds ratio; CI, confidence interval.

a Adjusted for sex, age, smoking and drinking status in logistic regression model

**Figure 2:**
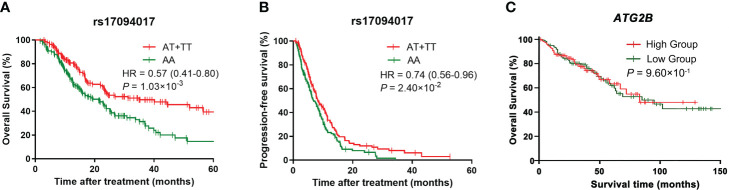
Kaplan-Meier curves of CRC patients. **(A–B)** Kaplan-Meier curves for overall survival **(A)** and progression-free survival **(B)** for rs17094017 in clinical patients with CRC by the Cox regression model. **(C)** Kaplan-Meier curve for *ATG2B* expression levels in the TCGA database by the Cox regression model.

Moreover, Kaplan-Meier curves were employed to verify the effects of genetic variants on *ATG2B* rs17094017 and CRC survival in the dominant model. The results revealed that *ATG2B* rs17094017 A > T might lead to a favorable prognosis (OS: HR = 0.57, 95% CI = 0.41-0.80, *P* = 1.03×10^-3^; PFS: HR = 0.74, 95% CI = 0.56-0.96, *P* = 2.40×10^-2^) (**Figures 2A, B**). Using the TCGA database, we compared the OS between *ATG2B* low-expressed group and high-expressed group. However, no significant difference was verified (*P* > 0.05) (**Figure 2C**).

### Stratified analyses of *ATG2B* rs17094017 in CRC prognosis

To compare the associations between clinical variables and CRC survival after therapy and *ATG2B* rs17094017, we performed stratification analyses in the dominant model, which included sex, age, cigarette smoking, alcohol intake, tumor site and grade, Dukes stage, metastases, and chemotherapy regimens.

**Figure 3:**
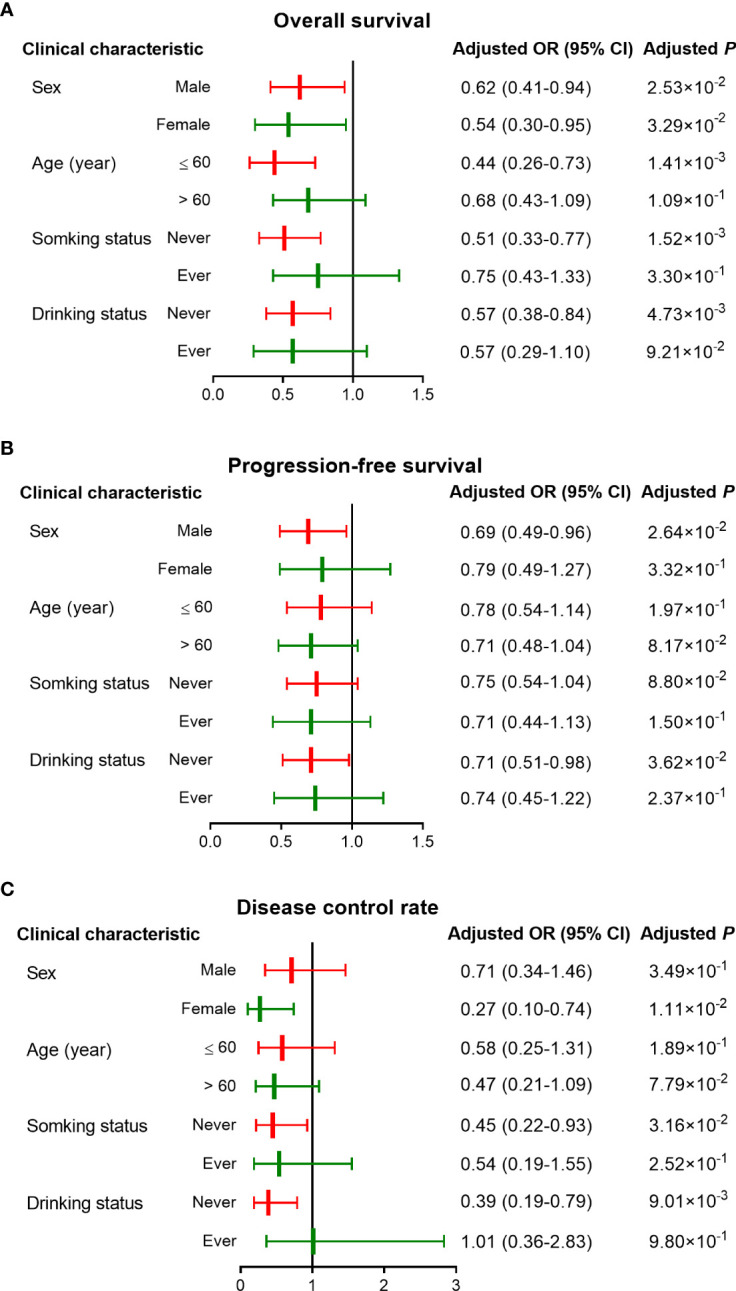
Stratified analyses of associations between rs17094017 and survival of clinical patients with CRC under the dominant model. **(A–B)** Stratified analyses of rs17094017 on overall survival **(A)** and progression-free survival **(B)**. **(C)** Stratified analyses of rs17094017 on disease control rate.

As **Figure 3** and **Table 4** shown, the AT/TT genotypes had significant associations with a longer OS time than genotype CC in the subgroups of well and moderate tumor grade (HR = 0.56, 95% CI = 0.37-0.82, d *P* = 3.32×10^-3^), Dukes stage D (HR = 0.60, 95% CI = 0.42-0.86, *P* = 5.00×10^-3^), younger age (HR = 0.44, 95% CI = 0.26-0.73, *P* = 1.41×10^-3^), non-smokers (HR = 0.51, 95% CI = 0.33-0.77, *P* = 1.52×10^-3^), and non-drinkers (HR = 0.57, 95% CI = 0.38-0.84, *P* = 4.73×10^-3^). Furthermore, the AT/TT genotype carriers had improved PFS and increased DCR in non-drinkers, colon cancer, and oxaliplatin-based chemotherapy subgroups (*P* < 0.05). In addition, rs17094017 was non-significant with PFS and DCR of CRC for subjects below 60. Therefore, there is no sufficient reason for age to be an influential factor in the prognosis of CRC.

**Table 4:** Stratified analysis of the association between rs17094017 and the survival of clinical patients with CRC in dominant model.

Clinical Characteristic	OS		PFS		DCR	
	HR (95% CI)^a^	*P* ^a^	HR (95% CI)^a^	*P* ^a^	OR (95% CI)^b^	*P* ^b^
Tumor site						
Colon	0.59 (0.38-0.91)	1.80×10^-2^	0.67 (0.47-0.95)	2.66×10^-2^	0.28 (0.13-0.60)	1.07×10^-3^
Rectum	0.55 (0.32-0.94)	2.76×10^-2^	0.90 (0.58-1.40)	6.45×10^-1^	1.37 (0.51-3.68)	5.37×10^-1^
Tumor grade						
Well and Moderate	0.56 (0.37-0.82)	3.32×10^-3^	0.72 (0.53-0.98)	3.48×10^-2^	0.67 (0.34-1.32)	2.48×10^-1^
Poor	0.67 (0.34-1.33)	2.49×10^-1^	0.81 (0.45-1.46)	4.87×10^-1^	0.20 (0.05-0.75)	1.67×10^-2^
Dukes stage						
C	2.70 (0.07-102.97)	5.93×10^-1^	0.58 (0.09-4.02)	5.86×10^-1^	NA	NA
D	0.60 (0.42-0.86)	5.00×10^-3^	0.76 (0.57-1.02)	6.45×10^-2^	0.58 (0.31-1.10)	9.35×10^-2^
Metastasis						
≤ 2	0.63 (0.43-0.93)	2.03×10^-2^	0.76 (0.56-1.03)	7.64×10^-2^	0.51 (0.26-1.01)	5.20×10^-2^
> 2	0.21 (0.08-0.52)	7.42×10^-4^	0.45 (0.19-1.05)	6.51×10^-2^	0.26 (0.04-1.56)	1.40×10^-1^
Chemotherapy						
Oxaliplatin	0.54 (0.34-0.87)	1.09×10^-2^	0.62 (0.43-0.90)	1.18×10^-2^	0.35 (0.15-0.82)	1.60×10^-2^
Irinotecan	0.61 (0.37-1.00)	4.92×10^-2^	0.87 (0.58-1.29)	4.74×10^-1^	0.78 (0.35-1.74)	5.47×10^-1^

OS, overall survival; PFS, progression-free survival; DCR, disease control rate; HR, hazard ratio; OR, odds ratio; CI, confidence interval.

a Adjusted for sex, age, smoking and drinking status in Cox regression model.

b Adjusted for sex, age, smoking and drinking status in logistic regression model.

Moreover, CRC prognosis was different among various chemotherapy regimens. Hence, we further used stratification analyses to evaluate whether rs17094017 had an effect on OS, PFS, and DCR of CRC patients with different chemotherapy regimens. The results documented that the AT genotype was relevant to improving prognosis (OS: HR = 0.55, 95% CI = 0.33-0.90, *P* = 1.70×10^-2^; PFS: HR = 0.63,95% CI = 0.43-0.93, *P* = 2.13×10^-2^) and increasing DCR (OR = 0.40, 95% CI = 0.16-0.97, *P =* 4.34×10^-2^) compared with AA genotype carriers receiving oxaliplatin-based chemotherapy (**Supplementary Table 6**). In the oxaliplatin-based chemotherapy subgroup, rs17094017 T allele prolonged PFS (HR = 0.72, 95% CI = 0.54-0.95, *P* = 2.15×10^-2^), OS (HR = 0.64, 95% CI = 0.44-0.94, *P* = 2.19×10^-2^), and increased DCR (OR = 0.39, 95% CI = 0.19-0.82, *P* = 1.26×10^-2^) under the additive model (**Supplementary Table 6**). Similarly, the results were discovered in the dominant model (OS: HR = 0.54, 95% CI = 0.34-0.87, *P* = 1.09×10^-2^; PFS: HR = 0.62, 95% CI = 0.43-0.90, *P* = 1.18×10^-2^; DCR; OR = 0.35, 95% CI = 0.15-0.82, *P* = 1.60×10^-2^) (**Supplementary Table 6** and **Supplementary Figure 2**). In the irinotecan-based chemotherapy subgroup of the dominant model, patients with AT/TT genotypes had a longer OS time (HR = 0.61, 95% CI = 0.37-1.00, *P* = 4.92×10^-2^) (**Supplementary Table 6** and **Supplementary Figure 2**).

### *ATG2B* expression analysis in CRC tissue

**Figure 4:**
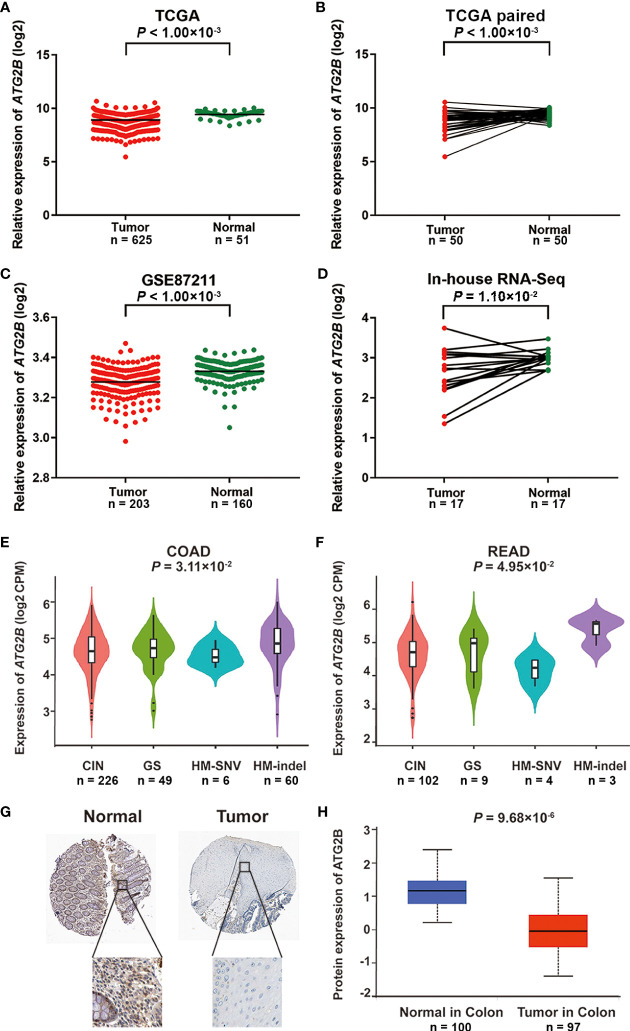
*ATG2B* expression in CRC and adjacent normal tissues. The mRNA expression of *ATG2B* in **(A)** unpaired tissues from the TCGA database, **(B)** paired tissues from the TCGA database, **(C)** GSE87211 database, and **(D)** in-house RNA-Seq data. *ATG2B* expression among various subtypes of **(E)** COAD and **(F)** READ in the TISIB database. **(G)** Images of ATG2B expression in normal and CRC tissues visualized by IHC in the HPA database. **(H)** The protein level of ATG2B in CRC tissues and normal tissues in the CPTAC database. CIN, chromosome instability; GS, Genome Stable; HM-SNV, Hypermutated - single nucleotide variants; HM-Indel, Hypermutated - insertion-deletion; COAD, colon adenocarcinoma; READ, rectal adenocarcinoma.

We then assessed the *ATG2B* mRNA expression in 17 paired clinical samples and validated the results in the TCGA and GEO datasets. As presented in **Figures 4A–D**, *ATG2B* was lower-expressed in tumor tissues than in noncancerous tissues of the subjects TCGA database, *P* < 1.00×10^-3^; TCGA paired data, *P* < 1.00×10^-3^; GSE87211, *P* < 1.00×10^-3^ and in-house RNA-Seq data, *P* = 1.10×10^-2^. The association between *ATG2B* mRNA expression and various subtypes of CRC was assessed in the TISIB database (colon adenocarcinoma, *P* = 3.11×10^-2^; rectal adenocarcinoma, *P* = 4.95×10^-2^) (**Figures 4 E, F****)**. A similar result was discovered at the protein expression level of ATG2B (**Figures 4 G, H****)**. Likewise, we compared the *ATG2B* mRNA expression in other tumors to adjacent normal tissues using the GEPIA online tool. *ATG2B* was lowly expressed in most tumor tissues. (**Supplementary Figure 3A**). Based on the Cancer Cell Line Encyclopedia, *ATG2B* expression seemed lower in CRC cells compared to other human cancer cell lines (**Supplementary Figure 3B**).

To further explore the relationships between clinical variables and mRNA expression of *ATG2B* in CRC tissues, we chose the characteristics of tumor stages, metastasis, sex, age, family history, tumor site, BMI, and *KRAS* mutation status for analysis in TCGA datasets. The results revealed that *ATG2B* mRNA expression at each stage in tumor tissues was significantly different from those in noncancerous tissues of the subjects (*P* < 1.00×10^-4^) (**Supplementary Figure 4A**). The mRNA expression of *ATG2B* in adjacent normal tissues was higher than in CRC tissues with or without metastasis (**Supplementary Figure 4B**). However, no differences were found in these clinical variables (*P* > 0.05) (**Supplementary Figures 4 C–H**).

### Functional prediction of *ATG2B*

To validate the relationship between the global gene-expression profile and ATG2B, we compared DEGs with low- and high-expressed *ATG2B* groups in the TCGA database. The results suggested that there was an up-regulation of 308 genes and a down-regulation of 369 genes (**Supplementary Figure 5A**). To further explore *ATG2B*-associated pathways, we analyzed significant differences between ATG2B-high and *ATG2B*-low expression groups using GSEA analysis in the *TCGA* datasets. *ATG2B* was significantly associated with inflammation and immune pathways, such as the Wnt signing pathway, tumor necrosis factor signing pathway, and T cell receptor signing pathway (**Supplementary Figure 5B**). As shown in **Supplementary Figure 6A**, *ATG2B* expression had a positive effect on dendritic cells, B cells, macrophages, CD8+ T cells, neutrophils, and CD4+ T cells in CRC by the TIMER database (P < 0.05). Furthermore, there were significant associations that were found in immune cell infiltration levels under various copy numbers of *ATG2B* both in colon adenocarcinoma and rectal adenocarcinoma (**Supplementary Figure 6B**).

## Discussion

In our study, the association between SNPs in autophagy-related genes and CRC outcome for chemotherapy treatment were assessed. *ATG2B* rs17094017 A > T had a significant effect on the prolonged OS and PFS of CRC with oxaliplatin-based chemotherapy. Interestingly, *ATG2B* rs17094017 T allele was associated with DCR in CRC patients. It was concluded that rs17094017 A > T in *ATG2B* might predict a better prognosis for CRC patients with oxaliplatin-based chemotherapy.

Chemotherapy is one of the primary treatments for CRC. However, there is a significant difference in individual responses to the efficacy of chemotherapy. Growing evidence revealed that autophagy played a great role in mediating resistance to chemotherapy. Melanoma patients who were resistant to the BRAF inhibitor showed higher levels of autophagy through the endoplasmic reticulum stress response (25). A study revealed that inhibition of intestinal epithelial autophagy through intestinal flora could improve CRC patients’ responses to chemotherapy and alter outcomes (26). In ovarian cancer, the resistance of the cytotoxic drug paclitaxel has been attributed to autophagy induction (27).

Autophagy-related gene variants were believed to be closely related to the development of cancers, and core genes were considered to affect the functions of the cells in metabolism, proliferation, apoptosis, and immunity (28, 29). Recently, the relationships between autophagy-related gene variants and cancer prognosis have been evaluated. For example, the association between rs473543 in *ATG5* and disease-free survival (DFS) of breast cancer patients undergoing chemotherapy was reported (30). Recent evidence also suggested a significant association between *ATG2B* rs17784271 and poor local recurrence-free survival and PFS in non-small cell lung cancer after radiotherapy (31). *ATG2B* rs3759601 has been shown to have therapeutic effects on bladder cancer treated with Bacillus Calmette-Guerin (16). A truncated variant of *UVRAG* was related to the transformation and tumor metastasis of CRC (32). *ATG16L1* T300A has an effect on a good prognosis in CRC (17). Moreover, *FIP200* rs1129660 played a crucial role in bevacizumab-mediated toxicity of mCRC (21). However, in the Chinese Han population, no study involving the relationships of SNPs in autophagy-related genes with CRC survival undergoing chemotherapy was found. Our study is the first to explore the association between *ATG2B* rs17094017 and the prognosis of CRC patients receiving chemotherapy among the Chinese population.

*ATG2B*, located on chromosome 14q32.2, is necessary for forming autophagosomes (33). The roles of ATG2A, ATG2B, and WIPI proteins are crucial for the membrane extension of the PI3P formation site in autophagy (12). It has been previously demonstrated that frameshift mutations of *ATG2B* with mononucleotide repeats occur in both CRC and gastric cancer (34). Additionally, our study revealed that *ATG2B* was lowly expressed in CRC tissues compared to noncancerous tissues. Reported research indicated that *ATG2B* displayed low expression in breast cancer (35). *ATG2B* was down-regulated in inflamed tissues compared to adjacent noninflamed tissues in Crohn’s disease (36). The downregulation of *ATG2B* activated cancer-associated fibroblasts (CAFs) by inhibiting autophagy in *P53*-deficient status, which accelerated the proliferation of CRC cells (37). Based on our results, the inhibition of autophagy promoted the development of cancer cells and affected chemosensitivity through suppressing *ATG2B* expression.

Moreover, autophagy plays an essential role in anti-tumor by regulating the immune system (38). Autophagy gene deficiency interferes with the survival, development, and differentiation of T and B cells (39, 40). Additionally, autophagy-mediated lipolysis inhibited the mitochondrial oxidative respiration pathway of neutrophil differentiation, which further affected immune defense mechanisms leading to cancer (41). Therefore, we further used GSEA analysis to predict the enrichment pathway of *ATG2B*. The results revealed that *ATG2B* was significantly related to the inflammatory and immune pathways. Based on TIMER databases, a positive correlation was discovered between *ATG2B* and tumor-infiltrating lymphocytes, suggesting that *ATG2B* might influence the immunotherapy of CRC modulating the tumor-infiltrating immune cells, but functional studies of *ATG2B* still need further verification.

CRC, a complex disease, which is ascribed to lifestyle and genetic factors (7). Hence, stratification analysis was used to obtain the association between clinical characteristics and CRC prognosis in this study. The results illustrated that rs17094017 was related to improved prognosis and increased DCR in colon cancer patients without drinking. A prospective cohort study suggested that patients with oxaliplatin-based chemotherapy had a longer OS time than those receiving irinotecan-based chemotherapy (42). In our study, we discovered that rs17094017 was meaningfully related to prolonged PFS, OS, and increased DCR in oxaliplatin-based chemotherapy rather than in irinotecan-based chemotherapy.

There are still several limitations to the research. Firstly, the sample size of the CRC population was relatively small. Thus, a larger population with more complete survival data is required to verify these results. Secondly, evidence concerning biological experiments with *ATG2B* is lacking. Hence, more basic biological research is required to further illustrate the effects of *ATG2B* in CRC.

Taken together, this study indicated that rs17094017 in *ATG2B* was related to a better outcome in CRC patients receiving chemotherapy. Furthermore, our study revealed that *ATG2B* rs17094017 was associated with increased DCR in CRC patients after treatment. SNP rs17094017 could serve as a novel biomarker to predict a CRC patient’s prognosis undergoing chemotherapy, providing a theoretical basis for individualized therapy in CRC. In summary, the association of genetic effects in *ATG2B* and the survival of CRC patients receiving chemotherapy was first explored among the Chinese population.

## Data availability statement

The original contributions presented in the study are included in the article/**Supplementary Material**. Further inquiries can be directed to the corresponding authors.

## Ethics statement

The studies involving human participants were reviewed and approved by The Institutional Review Board of Nanjing Medical University. The patients/participants provided their written informed consent to participate in this study.

## Author contributions

LZ, SL, and TC designed the study. TY and SB wrote the manuscript. SL critically revised the paper. SC assisted in the data analysis. LM, LJ, and YL collected the samples. All authors contributed to the article and approved the submitted version.

## Funding

Natural Science Foundation of Jiangsu Province (BK20201495), Jiangsu Provincial Medical Talent (ZDRCA2016089), and National Natural Science Foundation of China (No. 82102981) supported our research.

## Acknowledgments

We wish to thank Prof. Meilin Wang for providing data.

## Conflict of interest

The authors declare that the research was conducted in the absence of any commercial or financial relationships that could be construed as a potential conflict of interest.

## Publisher’s note

All claims expressed in this article are solely those of the authors and do not necessarily represent those of their affiliated organizations, or those of the publisher, the editors and the reviewers. Any product that may be evaluated in this article, or claim that may be made by its manufacturer, is not guaranteed or endorsed by the publisher.

## Abbreviations

CI, confidence interval; CPTAC, clinical proteomic tumor analysis consortium; CR, complete response; CRC, colorectal cancer; DCR, disease control rate; DEGs, differentially expressed genes; DFS, disease-free survival; FDR, false discovery rate; GEO, Gene Expression Omnibus; GEPIA, Gene Expression Profiling Interactive Analysis; GSEA, Gene Set Enrichment Analysis; HPA, Human Protein Atlas; HR, hazard ratio; HWE, Hardy-Weinberg equilibrium; LD, linkage disequilibrium; MAF, minor allele frequency; mCRC, metastatic colorectal cancer; OR, odds ratio; OS, overall survival; PD, progressive disease; PFS, progression free survival; PR, partial response; SD, stable disease; SNP, single-nucleotide polymorphisms; TCGA, The Cancer Genome Atlas; TIMER, Tumor Immune Estimation Resource; TISIDB, Tumor and Immune System Interaction Database.
