# Supplementary material for: Genetic variants in autophagy-related gene ATG2B predict the prognosis of colorectal cancer patients receiving chemotherapy
Source: Front Oncol. 2022 Aug 5;12:876424. doi: 10.3389/fonc.2022.876424 (PMC9389459; doi:10.3389/fonc.2022.876424)
Supplement: Supplementary file 1 [file DataSheet_1.docx]

**Supplementary Figure legends**

**Supplementary Figure 1.** **The protein-protein interaction (PPI) network of (A) autophagy-related genes and (B) the protein structure of ATG2B.**

**Supplementary Figure 2.** **Kaplan-Meier curves of CRC patients receiving oxaliplatin-based and irinotecan-based regimens.** (A-B) Kaplan-Meier curves for overall survival (A) and progression-free survival (B) for rs17094017 of CRC patients receiving oxaliplatin-based regimens by the Cox regression model. (C-D) Kaplan-Meier curves for overall survival (C) and progression-free survival (D) for rs17094017 of CRC patients receiving irinotecan-based regimens by the Cox regression model.

**Supplementary Figure 3. The expression levels of *ATG2B* (A) across all tumor tissues and normal tissues and (B) in 1457 human cancer cell lines from the CCLE database.** The height of the bar represents the median expression of a certain tumor type or normal tissue. ACC, Adrenocortical carcinoma; BLCA, Bladder Urothelial Carcinoma, BRCA, Breast invasive carcinoma; CESC, Cervical squamous cell carcinoma and endocervical adenocarcinoma; CHOL, Cholangio carcinoma; COAD, Colon adenocarcinoma; DLBC, Lymphoid Neoplasm Diffuse Large B-cell Lymphoma; ESCA, Esophageal carcinoma; GBM, Glioblastoma multiforme; HNSC, Head and Neck squamous cell carcinoma; KICH, Kidney Chromophobe; KIRC, Kidney renal clear cell carcinoma; KIRP, Kidney renal papillary cell carcinoma; LAML, Acute Myeloid Leukemia; LGG, Brain Lower Grade Glioma; LIHC, Liver hepatocellular carcinoma; LUAD, Lung adenocarcinoma; LUSC, Lung squamous cell carcinoma; OV, Ovarian serous cystadenocarcinoma; PAAD, Pancreatic adenocarcinoma; PCPG, Pheochromocytoma and Paraganglioma; PRAD, Prostate adenocarcinoma; READ, Rectum adenocarcinoma; SARC, Sarcoma; SKCM, Skin Cutaneous Melanoma; STAD, Stomach adenocarcinoma; TGCT, Testicular Germ Cell Tumors; THCA, Thyroid carcinoma; THYM, Thymoma; UCEC, Uterine Corpus Endometrial Carcinoma; UCS, Uterine Carcinosarcoma.

**Supplementary Figure 4. The expression levels of *ATG2B* in CRC tissues were stratified by subgroups.** (A) Tumor stages, (B) Tumor metastasis, (C) Sex, (D) Age, (E) Family history, (F) Tumor site, (G) BMI, and (H) *KRAS*. BMI, Body Mass Index, *****P* < 1.00×10^-4^.

**Supplementary Figure 5. Functional analyses of *ATG2B* in** **the the TCGA database.** (A) A volcano plot showing differentially expressed genes in high- and low-expressed *ATG2B* groups. (B) GSEA analysis of the differentially expressed genes between high- and low-expressed *ATG2B* groups in TCGA database.

**Supplementary Figure 6. The relationship of immune infiltration cells with *ATG2B* expression level in colorectal cancer.** (A) Associations between *ATG2B* expression and tumor purity, CD8+ T cells, CD4+ T cells, macrophages, neutrophils, and dendritic cells in COAD and READ based on the TIMER database. (B) The infiltration levels of various immune cells under different copy numbers of *ATG2B*. COAD colon adenocarcinoma; READ rectal adenocarcinoma.

**Supplementary Figure 1.**

**
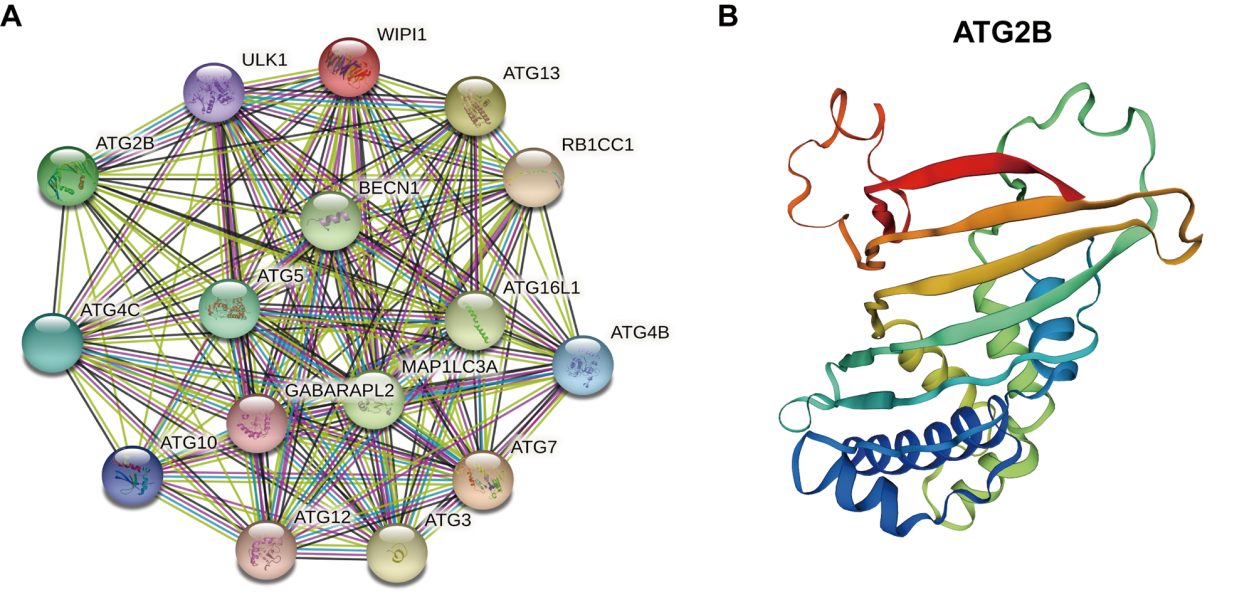
**

**Supplementary Figure 2.**

**
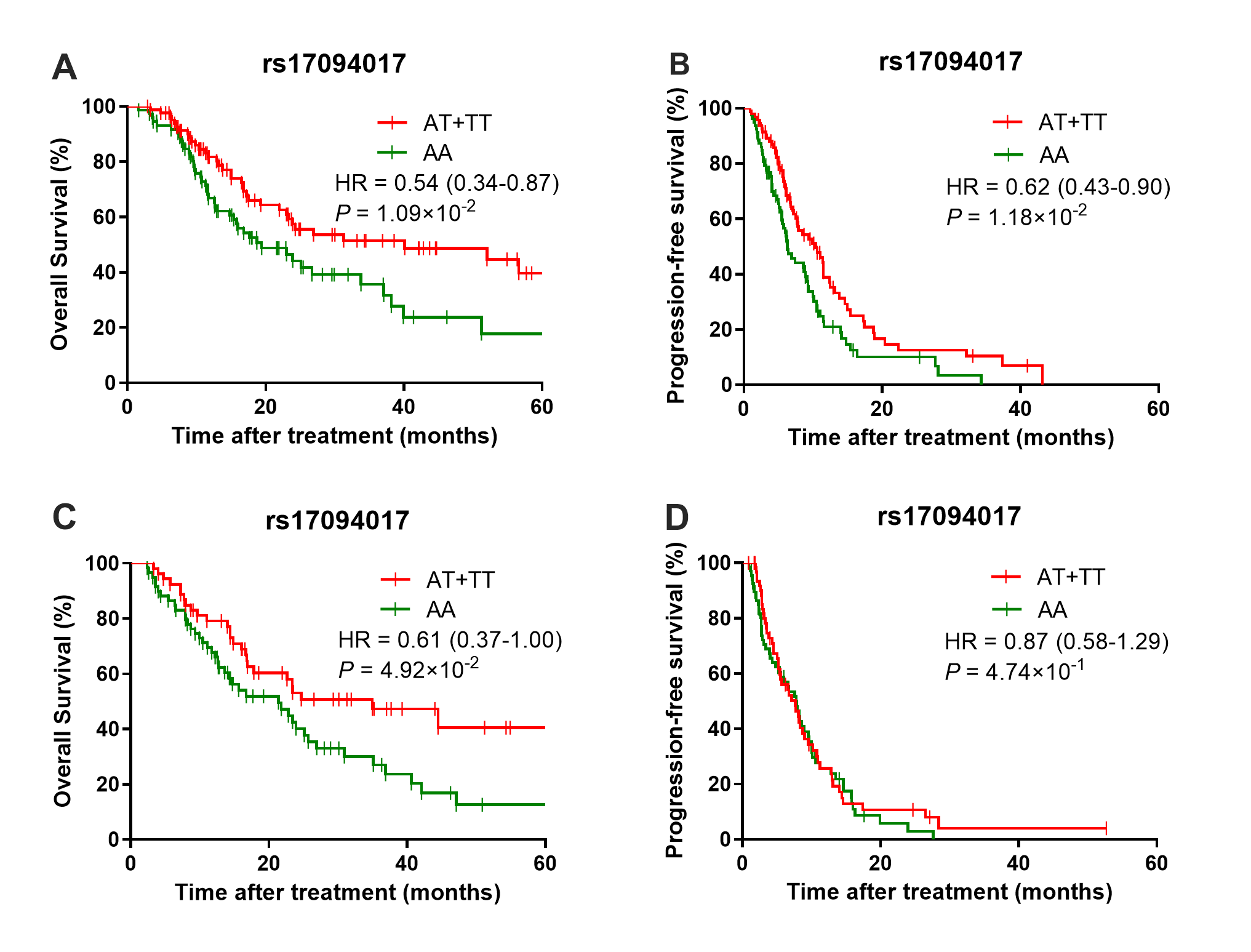
**

**Supplementary Figure 3.**

**
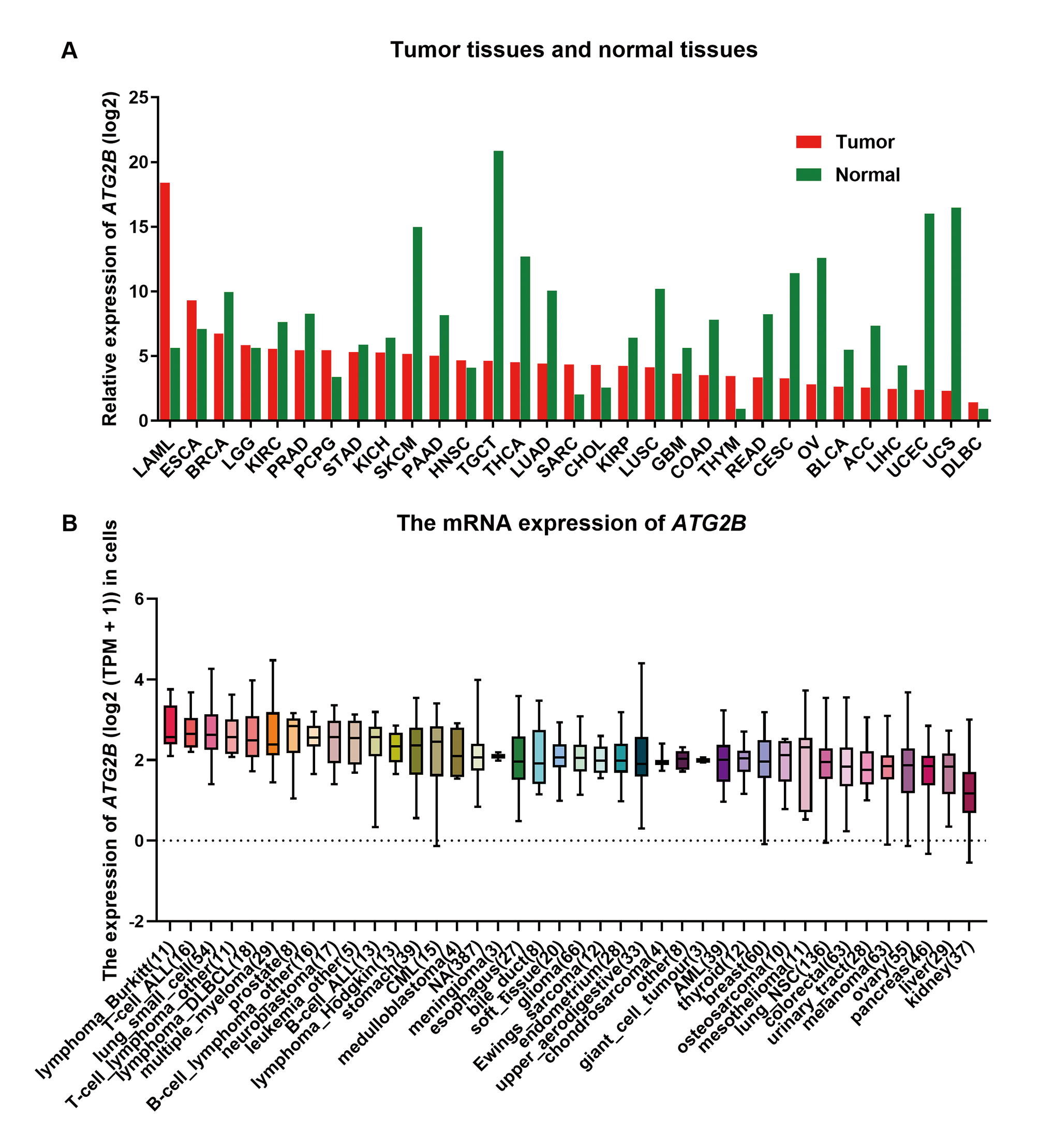
**

**Supplementary Figure 4.**

**
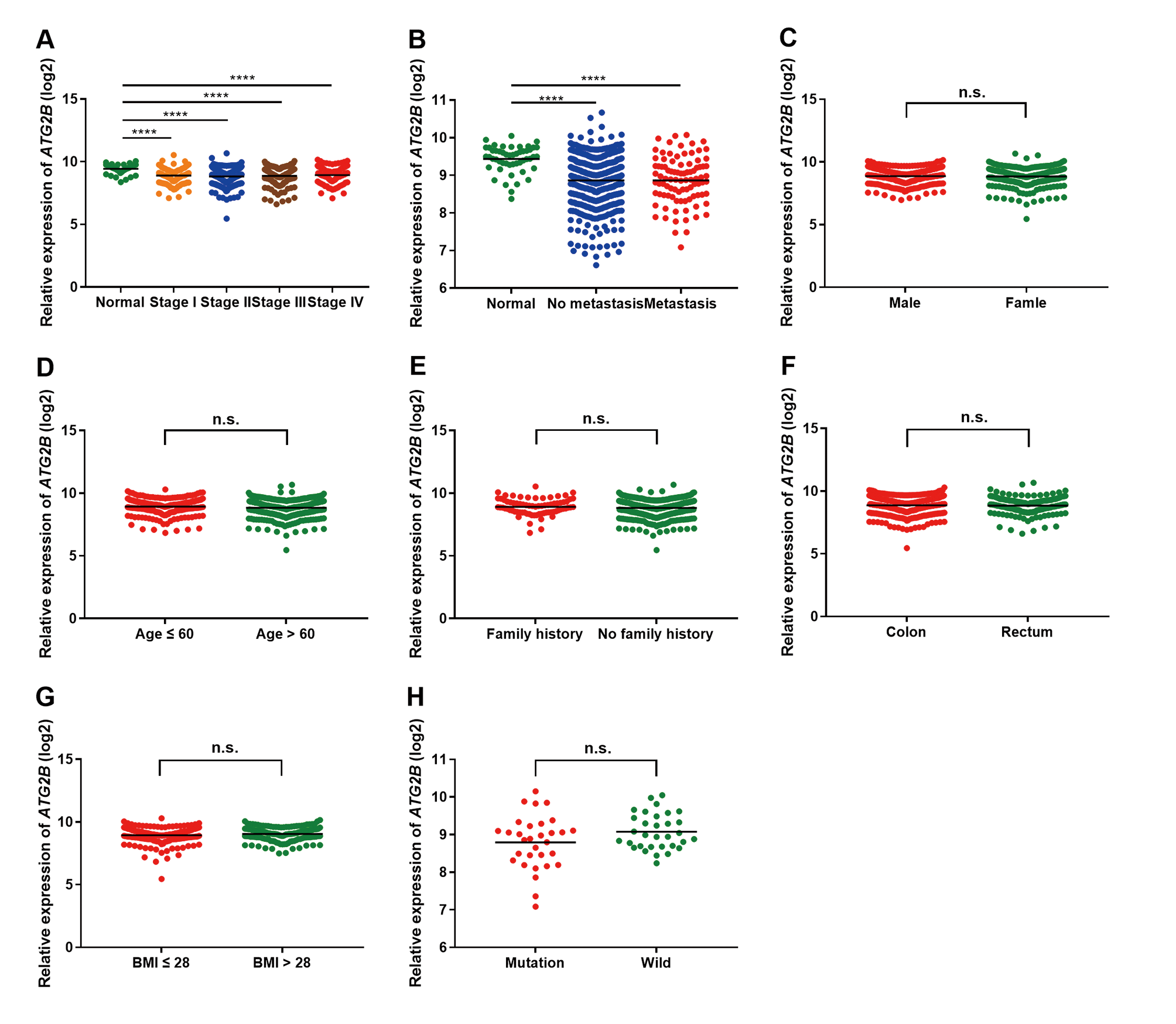
**

**Supplementary Figure 5.**


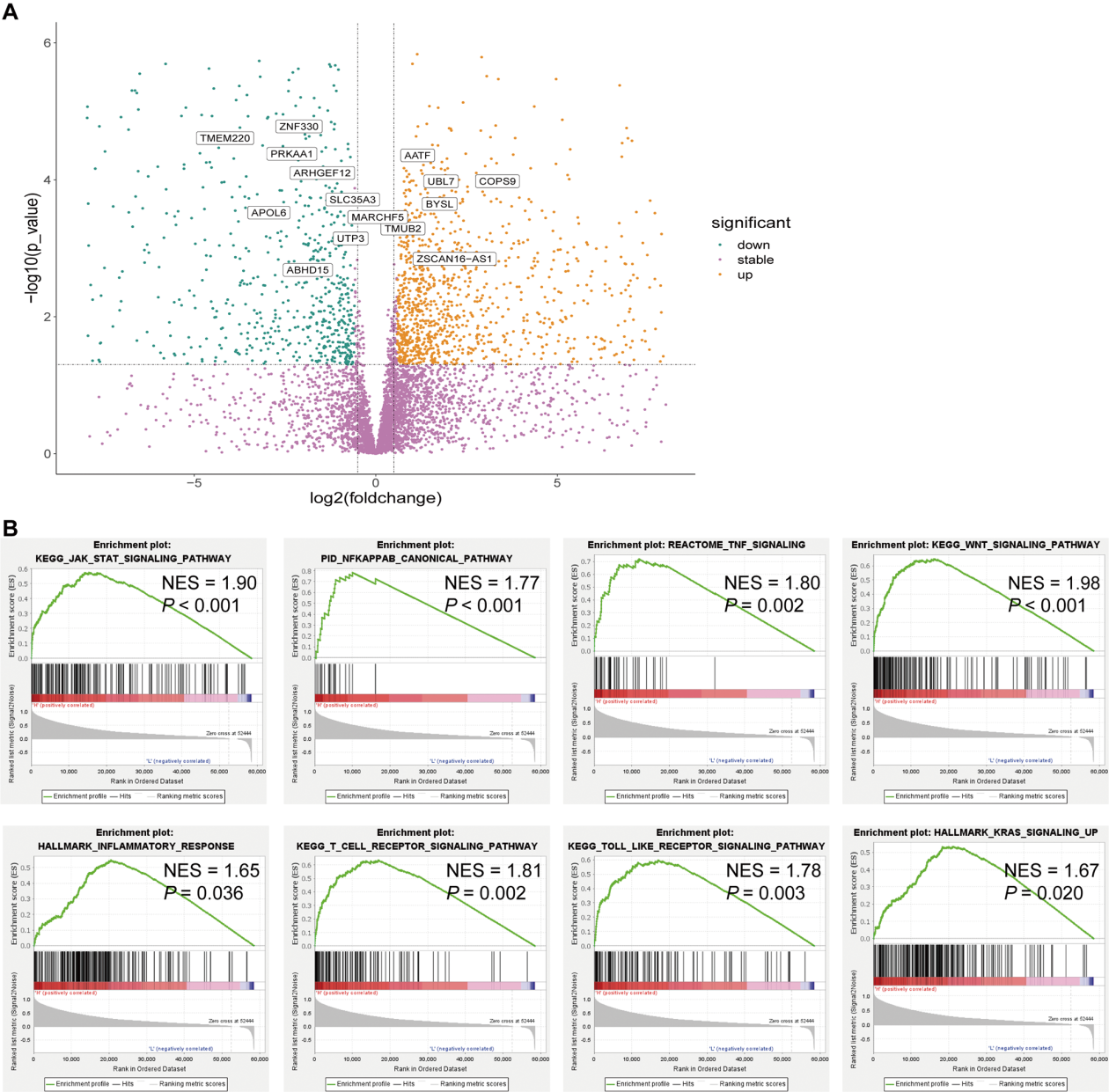


**Supplementary Figure 6.**


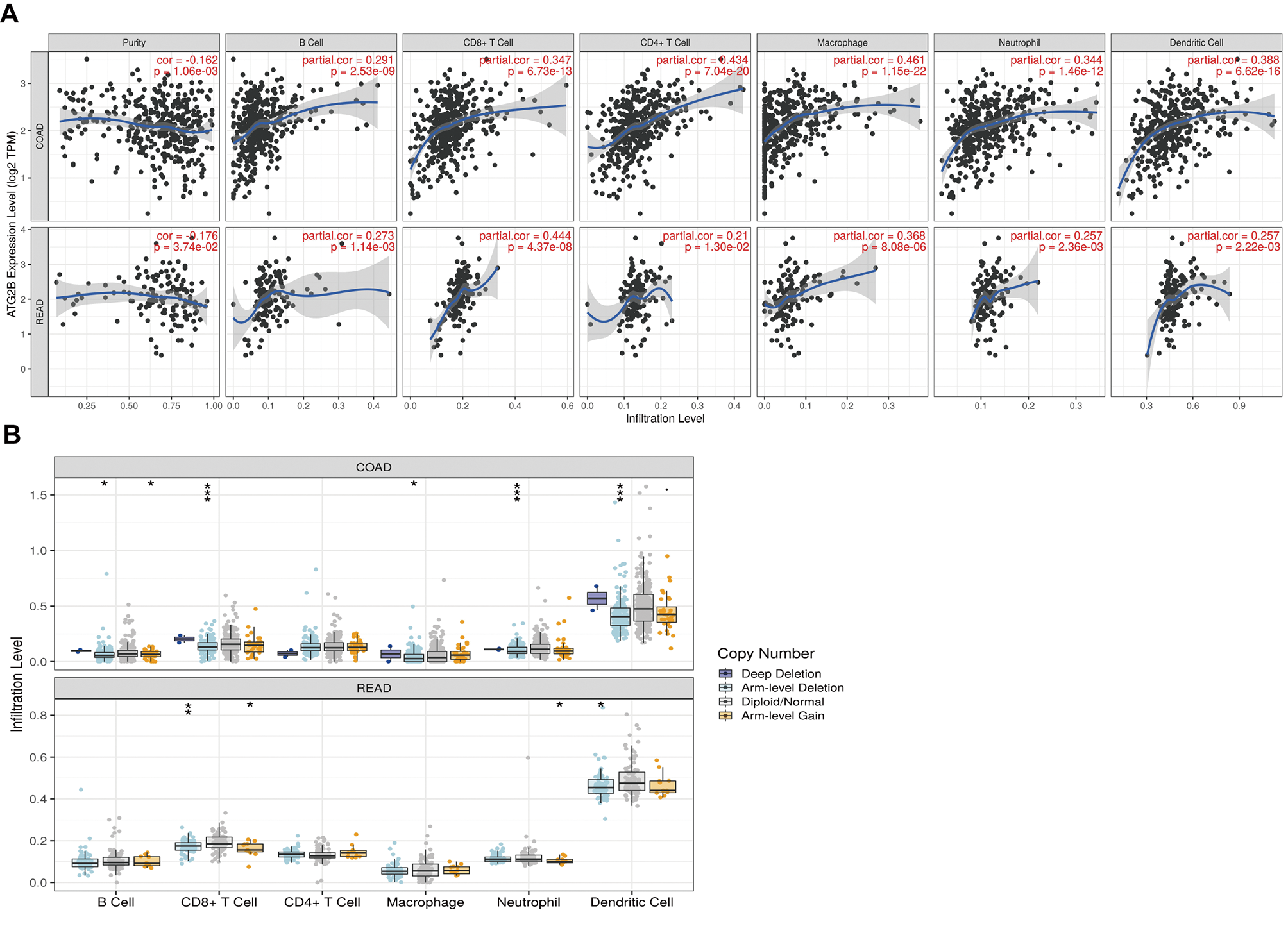


**Supplementary Table 1.** The characteristics and their association with overall survival of CRC patients.

| **Clinical characteristic** | **Patients (%)** | | ***P*^b^** | **HR (95% CI)** |
| --- | --- | --- | --- | --- |
| Sex |  |  | |  |
| Male | 205 (63.08) |  | | 1.00 |
| Female | 120 (36.92) | 0.519 | | 0.89 (0.63-1.26) |
| Age |  |  | |  |
| ≤ 60 | 175 (53.85) |  | | 1.00 |
| > 60 | 150 (46.15) | 0.401 | | 1.15 (0.83-1.58) |
| Tumor site |  |  | |  |
| Colon | 194 (59.69) |  | | 1.00 |
| Rectum | 131 (40.31) | 0.651 | | 1.08 (0.78-1.49) |
| Tumor grade |  |  | |  |
| Well and Moderate | 255 (78.46) |  | | 1.00 |
| Poor | 70 (21.54) | 0.084 | | 1.39 (0.96-2.00) |
| Dukes stage |  |  | |  |
| C | 23 (7.08) |  | | 1.00 |
| D | 302 (92.92) | 0.308 | | 0.67 (0.31-1.45) |
| Metastasis^a^ |  |  | |  |
| ≤ 2 | 237 (82.01) |  | | 1.00 |
| > 2 | 52 (17.99) | 0.070 | | 1.45 (0.97-2.16) |
| Chemotherapy |  |  | |  |
| Oxaliplatin | 188 (57.85) |  | | 1.00 |
| Irinotecan | 137 (42.15) | 0.242 | | 1.21 (0.88-1.67) |

HR, hazard ratio; CI, confidence interval.

^a^Some cases were not included due to missing metastasis details.

^b^Some cases were not included in Cox regression analyses due to missing clinical data or genotyping.

**Supplementary Table 2.** The key genes in the autophagy-related genes.

| **Chr** | **Gene** | **Description** |
| --- | --- | --- |
| 1 | *ATG4C* | autophagy related 4C cysteine peptidase |
| 2 | *ATG4B* | autophagy related 4B cysteine peptidase |
| 2 | *ATG16L1* | autophagy related 16 like 1 |
| 3 | *ATG3* | autophagy related 3 |
| 3 | *ATG7* | autophagy related 7 |
| 5 | *ATG10* | autophagy related 10 |
| 5 | *ATG12* | autophagy related 12 |
| 6 | *ATG5* | autophagy related 5 |
| 8 | *RB1CC1* | RB1 inducible coiled-coil 1 |
| 11 | *ATG13* | autophagy related 13 |
| 12 | *ULK1* | unc-51 like autophagy activating kinase 1 |
| 14 | *ATG2B* | autophagy related 2B |
| 16 | *GABARAPL2* | GABA type A receptor associated protein like 2 |
| 17 | *BECN1* | beclin 1 |
| 17 | *WIPI1* | WD repeat domain, phosphoinositide interacting 1 |
| 20 | *MAP1LC3A* | microtubule associated protein 1 light chain 3 alpha |

**Supplementary Table 3.** Expression of candidate genes in TCGA database.

| **Chr** | **Gene** | **Fold change** | ***P*** | **Call rate (tumor)** | **Call rate (normal)** |
| --- | --- | --- | --- | --- | --- |
| 1 | *ATG4C* | -1.29 | 1.00×10^-6^ | 1.00 | 1.00 |
| 2 | *ATG4B* | 1.26 | 1.68×10^-11^ | 1.00 | 1.00 |
| 2 | *ATG16L1* | 1.24 | 2.81×10^-20^ | 1.00 | 1.00 |
| 6 | *ATG5* | -1.22 | 8.00×10^-6^ | 1.00 | 1.00 |
| 14 | *ATG2B* | -1.40 | 2.00×10^-12^ | 1.00 | 1.00 |
| 16 | *GABARAPL2* | -1.35 | 6.76×10^-10^ | 1.00 | 1.00 |
| 17 | *BECN1* | -1.27 | 3.83×10^-22^ | 1.00 | 1.00 |
| 17 | *WIPI1* | -1.32 | 4.07×10^-8^ | 1.00 | 1.00 |

**Supplementary Table 4.** The function of 19 SNPs *in silico* analysis.

| **Chr** | **SNP** | **Gene** | **Allele^a^** | **Functional prediction** | | |
| --- | --- | --- | --- | --- | --- | --- |
|  |  |  |  | **Score^b^** | **HaploReg^c^** | **SNP info^d^** |
| 2 | rs35271226 | *ATG4B* | A/G | 4 | Enhancer histone marks, Proteins, Motifs changed, Selected eQTL hits | TFBS |
| 2 | rs1130910 | *ATG4B* | G/C | 4 | Enhancer histone marks, Motifs changed, Selected eQTL hits |  |
| 2 | rs7421 | *ATG4B* | C/T | 2b | DNAse, Motifs changed, Selected eQTL hits |  |
| 2 | rs6758317 | *ATG16L1* | T/C | 3a | Enhancer histone marks, Motifs changed, GRASP QTL hits, Selected eQTL hits |  |
| 2 | rs34691302 | *ATG4B* | T/C | 2b | Promoter, Enhancer histone marks, DNAse, Motifs changed, Selected eQTL hits | TFBS |
| 2 | rs2241878 | *ATG16L1* | C/T | 6 | Motifs changed, Selected eQTL hits | TFBS |
| 2 | rs7595748 | *ATG16L1* | A/G | 6 | Motifs changed, Selected eQTL hits | TFBS |
| 14 | rs17094017 | *ATG2B* | T/A | 6 | Motifs changed, Selected eQTL hits |  |
| 14 | rs8019013 | *ATG2B* | T/C | 5 | Enhancer histone marks, DNAse, Motifs changed, GRASP QTL hits, Selected eQTL hits |  |
| 14 | rs12432561 | *ATG2B* | A/G | 6 | Motifs changed, Selected eQTL hits |  |
| 14 | rs10134160 | *ATG2B* | T/C | 4 | DNAse, Proteins, Motifs changed, GRASP QTL hits, Selected eQTL hits |  |
| 16 | rs11149841 | *GABARAPL2* | T/G | 1f | Enhancer histone marks, DNAse, Motifs changed, GRASP QTL hits, Selected eQTL hits |  |
| 16 | rs6564267 | *GABARAPL2* | T/G | 1f | Enhancer histone marks, Motifs changed, GRASP QTL hits, Selected eQTL hits |  |
| 17 | rs11658979 | *WIPI1* | G/A | 2b | Enhancer histone marks, DNAse, Motifs changed, GRASP QTL hits, Selected eQTL hits |  |
| 17 | rs11077558 | *WIPI1* | C/G | 1b | Enhancer histone marks, DNAse, Proteins, Motifs changed, GRASP QTL hits, Selected eQTL hits |  |
| 17 | rs2011143 | *WIPI1* | T/C | 2b | Motifs changed, Selected eQTL hits | TFBS |
| 17 | rs2909207 | *WIPI1* | T/C | 1f | Enhancer histone marks, DNAse, Motifs changed, GRASP QTL hits, Selected eQTL hits |  |
| 17 | rs883622 | *WIPI1* | G/A | 3a | Enhancer histone marks, Motifs changed, Selected eQTL hits |  |
| 17 | rs883620 | *WIPI1* | C/G | 5 | Enhancer histone marks, DNAse, Motifs changed, Selected eQTL hits |  |

Chr, chromosome; SNP, single nucleotide polymorphism; DNAse, Deoxyribonuclease; TFBS, Transcription factors binding sites.

^a^Effect allele/reference allele.

^b^Based on Regulome DB (http://regulome.stanford.edu/).

^c^Based on HaploReg (http://compbio.mit.edu/HaploReg).

^d^Based on SNPinfo Web Server (http://snpinfo.niehs.nih.gov/).

**Supplementary Table 5.** Association of 19 selected SNPs and colorectal cancer PFS and DCR in additive model.

| **Chr** | **SNP** | **Gene** | **Allele^a^** | **PFS** | | **DCR** | |
| --- | --- | --- | --- | --- | --- | --- | --- |
|  |  |  |  | **HR (95% CI)^b^** | ***P*^b^** | **OR (95% CI)^c^** | ***P*^c^** |
| 2 | rs35271226 | *ATG4B* | A/G | 1.05 (0.87-1.26) | 6.12×10^-1^ | 1.54 (1.03-2.29) | 3.49×10^-2^ |
| 2 | rs1130910 | *ATG4B* | G/C | 0.93 (0.74-1.17) | 5.43×10^-1^ | 0.60 (0.35-1.04) | 6.71×10^-2^ |
| 2 | rs7421 | *ATG4B* | C/T | 1.18 (0.97-1.45) | 9.98×10^-2^ | 1.92 (1.25-2.96) | 2.87×10^-3^ |
| 2 | rs6758317 | *ATG16L1* | T/C | 0.81 (0.58-1.13) | 2.16×10^-1^ | 1.34 (0.71-2.55) | 3.66×10^-1^ |
| 2 | rs34691302 | *ATG4B* | T/C | 1.11 (0.77-1.59) | 5.85×10^-1^ | 1.40 (0.68-2.87) | 3.60×10^-1^ |
| 2 | rs2241878 | *ATG16L1* | C/T | 0.99 (0.81-1.22) | 9.46×10^-1^ | 0.88 (0.58-1.34) | 5.51×10^-1^ |
| 2 | rs7595748 | *ATG16L1* | A/G | 1.02 (0.84-1.23) | 8.54×10^-1^ | 0.86 (0.58-1.29) | 4.63×10^-1^ |
| 14 | rs17094017 | *ATG2B* | T/A | 0.76 (0.62-0.93) | 7.34×10^-3^ | 0.60 (0.37-0.96) | 3.31×10^-2^ |
| 14 | rs8019013 | *ATG2B* | T/C | 1.30 (1.05-1.60) | 1.43×10^-2^ | 1.45 (0.96-2.19) | 7.93×10^-2^ |
| 14 | rs12432561 | *ATG2B* | A/G | 1.02 (0.81-1.27) | 8.94×10^-1^ | 0.91 (0.58-1.42) | 6.68×10^-1^ |
| 14 | rs10134160 | *ATG2B* | T/C | 1.22 (0.95-1.57) | 1.27×10^-1^ | 0.79 (0.46-1.36) | 3.90×10^-1^ |
| 16 | rs11149841 | *GABARAPL2* | T/G | 1.08 (0.75-1.56) | 6.68×10^-1^ | 0.88 (0.43-1.84) | 7.38×10^-1^ |
| 16 | rs6564267 | *GABARAPL2* | T/G | 0.97 (0.68-1.37) | 8.48×10^-1^ | 0.98 (0.48-2.00) | 9.62×10^-1^ |
| 17 | rs11658979 | *WIPI1* | G/A | 1.06 (0.78-1.44) | 7.18×10^-1^ | 1.22 (0.68-2.21) | 5.05×10^-1^ |
| 17 | rs11077558 | *WIPI1* | C/G | 1.04 (0.86-1.25) | 7.08×10^-1^ | 1.16 (0.77-1.72) | 4.80×10^-1^ |
| 17 | rs2011143 | *WIPI1* | T/C | 1.16 (0.95-1.41) | 1.45×10^-1^ | 1.11 (0.73-1.67) | 6.27×10^-1^ |
| 17 | rs2909207 | *WIPI1* | T/C | 1.05 (0.87-1.26) | 6.27×10^-1^ | 1.08 (0.73-1.60) | 6.89×10^-1^ |
| 17 | rs883622 | *WIPI1* | G/A | 0.88 (0.72-1.08) | 2.22×10^-1^ | 0.87 (0.56-1.34) | 5.24×10^-1^ |
| 17 | rs883620 | *WIPI1* | C/G | 0.90 (0.60-1.37) | 6.33×10^-1^ | 0.77 (0.32-1.87) | 5.69×10^-1^ |

Chr, chromosome; SNP, single nucleotide polymorphism; PFS, progression-free survival; DCR, disease control rate; HR, hazard ratio; OR, odds ratio; CI, confidence interval.

^a^Effect allele/reference allele.

^b^For additive model adjusted for sex, age, smoking and drinking status in Cox regression model.

^c^For additive model adjusted for sex, age, smoking and drinking status in logistic regression model.

**Supplementary Table 6.** Association between rs17094017 and OS, PFS and DCR in analysis stratified by chemotherapy.

| **Chemotherapy** | **OS** | | **PFS** | | **DCR** | |
| --- | --- | --- | --- | --- | --- | --- |
|  | **HR (95% CI)^a^** | ***P*^a^** | **HR (95% CI)^a^** | ***P*^a^** | **OR (95% CI)^b^** | ***P*^b^** |
| Oxaliplatin |  |  |  |  |  |  |
| AA | 1.00 |  | 1.00 |  | 1.00 |  |
| AT | 0.55 (0.33-0.90) | 1.70×10^-2^ | 0.63 (0.43-0.93) | 2.13×10^-2^ | 0.40 (0.16-0.97) | 4.34×10^-2^ |
| TT | 0.53 (0.23-1.21) | 1.30×10^-1^ | 0.59 (0.32-1.09) | 9.35×10^-2^ | 0.15 (0.02-1.21) | 7.46×10^-2^ |
| Additive model | 0.64 (0.44-0.94) | 2.19×10^-2^ | 0.72 (0.54-0.95) | 2.15×10^-2^ | 0.39 (0.19-0.82) | 1.26×10^-2^ |
| Dominant model | 0.54 (0.34-0.87) | 1.09×10^-2^ | 0.62 (0.43-0.90) | 1.18×10^-2^ | 0.35 (0.15-0.82) | 1.60×10^-2^ |
| Recessive model | 0.72 (0.33-1.60) | 4.23×10^-1^ | 0.75 (0.42-1.35) | 3.39×10^-1^ | 0.23 (0.03-1.82) | 1.63×10^-1^ |
| Irinotecan |  |  |  |  |  |  |
| AA | 1.00 |  | 1.00 |  | 1.00 |  |
| AT | 0.60 (0.35-1.01) | 5.68×10^-2^ | 1.01 (0.67-1.53) | 9.56×10^-1^ | 0.76 (0.33-1.76) | 5.24×10^-1^ |
| TT | 0.65 (0.23-1.84) | 4.13×10^-1^ | 0.39 (0.15-1.02) | 5.39×10^-2^ | 0.93 (0.17-5.19) | 9.30×10^-1^ |
| Additive model | 0.68 (0.45-1.04) | 7.83×10^-2^ | 0.80 (0.59-1.09) | 1.53×10^-1^ | 0.85 (0.43-1.66) | 6.31×10^-1^ |
| Dominant model | 0.61 (0.37-1.00) | 4.92×10^-2^ | 0.87 (0.58-1.29) | 4.74×10^-1^ | 0.78 (0.35-1.74) | 5.47×10^-1^ |
| Recessive model | 0.79 (0.28-2.21) | 6.48×10^-1^ | 0.39 (0.15-1.00) | 5.00×10^-2^ | 1.04 (0.19-5.63) | 9.64×10^-1^ |

OS,overall survival; PFS, progression-free survival; DCR, disease control rate; HR, hazard ratio; OR, odds ratio; CI, confidence interval.

^a^Adjusted for sex, age, smoking and drinking status in Cox regression model.

^b^Adjusted for sex, age, smoking and drinking status in logistic regression model.
